# Identification of a shared gene signature and biological mechanism between diabetic foot ulcers and cutaneous lupus erythemnatosus by transcriptomic analysis

**DOI:** 10.3389/fphys.2024.1297810

**Published:** 2024-02-16

**Authors:** Siqi Wu, Yuetong Wang, Jingyi Duan, Ying Teng, Dali Wang, Fang Qi

**Affiliations:** ^1^ Department of Burns and Plastic Surgery, Affiliated Hospital of Zunyi Medical University, Zunyi, China; ^2^ The Collaborative Innovation Center of Tissue Damage Repair and Regeneration Medicine of Zunyi Medical University, Zunyi, China; ^3^ Faculty of Medicine, Dentistry and Health Sciences, The University of Melbourne, Parkville, VIC, Australia; ^4^ Medicine and Technology College of Zunyi Medical University, Zunyi, China

**Keywords:** diabetic foot ulcer, DFU, cutaneous lupus erythematosus, CLE, bioinformatics, common key genes, inflammatory

## Abstract

Diabetic foot ulcers (DFU) and cutaneous lupus erythematosus (CLE) are both diseases that can seriously affect a patient’s quality of life and generate economic pressure in society. Symptomatically, both DLU and CLE exhibit delayed healing and excessive inflammation; however, there is little evidence to support a molecular and cellular connection between these two diseases. In this study, we investigated potential common characteristics between DFU and CLE at the molecular level to provide new insights into skin diseases and regeneration, and identify potential targets for the development of new therapies. The gene expression profiles of DFU and CLE were obtained from the Gene Expression Omnibus (GEO) database and used for analysis. A total of 41 common differentially expressed genes (DEGs), 16 upregulated genes and 25 downregulated genes, were identified between DFU and CLE. GO and KEGG analysis showed that abnormalities in epidermal cells and the activation of inflammatory factors were both involved in the occurrence and development of DFU and CLE. Protein-protein interaction network (PPI) and sub-module analysis identified enrichment in seven common key genes which is *KRT16*, *S100A7*, *KRT77, OASL, S100A9, EPGN* and *SAMD9*. Based on these seven key genes, we further identified five miRNAs(has-mir-532-5p, has-mir-324-3p,has-mir-106a-5p,has-mir-20a-5p,has-mir-93-5p) and7 transcription factors including CEBPA, CEBPB, GLI1, EP30D, JUN,SP1, NFE2L2 as potential upstream molecules. Functional immune infiltration assays showed that these genes were related to immune cells. The CIBERSORT algorithm and Pearson method were used to determine the correlations between key genes and immune cells, and reverse key gene-immune cell correlations were found between DFU and CLE. Finally, the DGIbd database demonstrated that Paquinimod and Tasquinimod could be used to target S100A9 and Ribavirin could be used to target OASL. Our findings highlight common gene expression characteristics and signaling pathways between DFU and CLE, indicating a close association between these two diseases. This provides guidance for the development of targeted therapies and mutual interactions.

## 1 Introduction

Diabetic foot ulcer (DFU) is one of the most serious complications of diabetes mellitus and presents as chronic, non-healing wounds caused by diabetic sensory, motor, and autonomic neuropathy, vascular disease, and bacterial infection (Armstrong et al., 2023). Up to approximately 34% of individuals with diabetes will develop a foot ulcer during their lifetime, and the mortality rate at 5 years for patients with DFU is 2.5-fold higher than the risk for patients with diabetes who do not suffer from foot ulcers (Walsh et al., 2016; Armstrong et al., 2017). DFU causes significant financial strain on patients and is associated with high medical costs. Investigating the pathogenesis of DFU and potential targets is an immense challenge in the field of wound healing and tissue regeneration.

Systemic lupus erythematosus (SLE) is a chronic autoimmune disease that is affected by gender, race, genetics and other factors. This condition can affect multiple organ systems across the body; of these, the skin is the second most affected organ (Lee and Sinha, 2006). It is estimated that approximately 70% of SLE cases involve cutaneous manifestations, known as cutaneous lupus erythematosus (CLE); however, there are currently no FDA-approved treatments for CLE (Tsoi et al., 2019).

Skin lesions are a common symptom of DFU and CLE, and both of these conditions usually present with persistent, non-healing wounds (Doersch et al., 2017; Zubair and Ahmad, 2019). DFU patients suffer from lesions and abrasions with the loss of epithelial cells; these lesions and abrasions may extend to the dermis and deeper layers, even to the bone and muscle (Zubair and Ahmad, 2019). This condition can manifest as hyperkeratosis and necrotic dermal tissue, deep tissue abscess, and gangrene (Lebrun et al., 2010). CLE patients also suffer from several clinical manifestations, including epidermal atrophy, hyperkeratosis, inflammation at the dermal-epidermal junction, rash and erythema (Lee and Sinha, 2006). However, evidence relating to the specific characterization of these common traits is scarce, especially in terms of cellular and molecular mechanisms.

It is widely recognized that the immune environment of DFU is atypical and involves a perpetual inflammatory response. Analysis of gene expression in CLE skin samples and blood samples also revealed over-activation of the innate immune response pathway (Scholtissek et al., 2017; Zhu et al., 2021). Abnormal macrophage polarization is a primary cause of delayed healing in patients with DFU (Huang et al., 2021), and the skin afflicted by CLE is known to have higher expression levels of M1 macrophage-related proteins (Chong et al., 2015). Epidermal damage and hyperkeratosis are also the clinical manifestations of DFU and CLE (Lebrun et al., 2010; Doersch et al., 2017; Zubair and Ahmad, 2019). According to previous studies, the apoptosis of keratinocytes can be detected in the lesions of most patients with CLE (Järvinen et al., 2007). It is also possible that keratinocytes participate in the pathological process of CLE by releasing the production of proinflammatory cytokines (Zhang et al., 2017). However, in DFU patients, hyperglycemia can alter a number of key mechanisms and also lead to reduced keratinocyte proliferation and migration (Hosseini Mansoub, 2021). Overall, there are some similarities in the molecular features of DFU and CLE, although the specific associations and potential for crosstalk remain largely unclear. It is crucial to determine the common molecular relationship between DFU and CLE in terms of pathogenesis and progression if we are to provide efficient diagnoses and therapeutic interventions.

The utilization of high-throughput sequencing technology and bioinformatics is providing us with an effective tool with which to investigate the association between diseases. In the present study, we used bioinformatics methods to investigate the key genes and pathways that are common to DFU and CLE. Assessment of the immune landscape revealed similar immune signatures and a transcription factor (TF)-microRNA (miRNA)-target network while drug prediction was used to evaluate the key function and therapeutic potentiality of key gene targets. This research provides a deeper understanding of the pathophysiological processes that may link DFU and CLE, thus providing novel strategies for future diagnosis and treatment in the clinic.

## 2 Materials and methods

### 2.1 Raw data collection

First, we obtained the gene expression profiles of DFU and CLE by searching the Gene Expression Omnibus (GEO) (Barrett et al., 2007) (https://www.ncbi.nlm.nih.gov/) database for “diabetic toot ulcer” and “cutaneous lupus erythematosus”. Then, the data identified (GSE134431, GSE80178, GSE81071 and GSE95474) were downloaded from the GEO, preprocessed, normalized and log2 transformed into a probe expression matrix. Then, we downloaded the annotation file from the GEO platform. By one-to-one matching between probe ID and gene symbol, probes that did not match the gene symbol were removed. In cases where different probes corresponded to the same gene, the mean value of the different probes was taken as the final expression value of the gene. Expression values and grouping information are shown in Supplementary Table S1.

### 2.2 Identification of DEGs

Differentially expressed genes (DEGs) were identified by applying the Bayesian method in the Limma package (Smyth, 2005) (Version 3.10.3, http://www.bioconductor.org/packages/2.9/bioc/html/limma.html). Differences in gene expression between the two diseases were investigated by comparing the DFU experimental group (GSE134431) and the CLE experimental group (GSE81071); corresponding *p*-values and logFC values were obtained for all genes. To eliminate false positive results from the GEO dataset, the *p*-value was adjusted (to generate an adj.*p*-value). Differentially expressed genes (DEGs) were identified by applying a specific threshold: an adj.*p*-value < 0.05 and a | logFC |<0.585. Then, we took the intersection of the two genes to identify specific genes that were either up-or downregulated in both diseases, thus representing the common genes.

### 2.3 Enrichment analysis of common DEGs

Next, we used the R package clusterProfilter (Yu et al., 2012) to perform Gene Ontology (GO) (Ashburner et al., 2000) and Kyoto Encyclopedia of Genes Genomes (KEGG) (Kanehisa, 2000) pathway enrichment analysis on the DEGs identified earlier. *p*-values < 0.05 were considered as significant enrichment.

### 2.4 PPI network construction and module analysis

The STRING database (Szklarczyk et al., 2015) (Version: 10.0, http://www.string-db.org/) was used to predict and analyze whether there was a mutual relationship between the proteins encoded by common DEGs. The input gene set included the common DEGs and the species was set to “*Homo sapiens*”. The parameter PPI score was set to 0.15; this required that the interacting protein nodes were all included in common DEGs. Then we used Cytoscape (Shannon et al., 2003) (version 3.4.0, http://chianti.ucsd.edu/cytoscape-3.4.0/) to construct a network diagram of the PPI relationship and generate a PPI network. We applied the MCODE (Bader and Hogue, 2003) (version 1.5.1, http://apps.cytoscape.org/apps/mcode) plugin with parameters set to default (Degree Cutoff = 2; Node Score Cutoff = 0.2; K-score = 2; Max Depth = 100) to identify genes in the network module and the sub-network diagrams of each module. Scores are given based on the importance of submodules. Finally, we analyzed the topological properties of nodes in the network by CytoNCA(Tang et al., 2015) (Version 2.1.6, http://apps.cytoscape.org/apps/cytonca), including four attributes: Degree, Edge Percolated Component (EPC), Maximal Clique Centrality (MCC) and Maximum Neighborhood Component (MNC). The larger the value of each attribute, the greater the role of the gene in the network. We selected the top 20 genes under each attribute in turn, and the genes obtained by intersection were used for further analysis.

### 2.5 Selection and analysis of key genes

The R package clusterProfiler (Yu et al., 2012) was used to perform GO (Ashburner et al., 2000), BP and KEGG (Kanehisa, 2000) pathway enrichment analysis of the key genes; *p* < 0.05 was considered a significant result. Then, we generated a box plot to show the distribution of expression for these key genes, as determined by PPI topology analysis of the validation set. Then, we used the *t*-test to calculate significance, and identify genes with significant differences (up- and downregulation) between the validation sets of the two diseases, thus generating a list of validation genes.

### 2.6 Assessment of the immune landscape

The immune response is involved in the progression of both DFU and CLE. To investigate whether the key genes identified herein were involved in the immune response, we determined correlations between the key genes and immune cell infiltration. First, the CIBERSORT (Chen et al., 2018) algorithm was used to calculate data relating to DFU and CLE and determine the proportions of 22 types of immune cells in each sample. Next, the correlation coefficients and *p*-values between key genes and each immune cell were calculated by Pearson’s method. Finally, we plotted a correlation heatmap. Then, we generated a scatterplot showing the immune cells and gene pairs with the highest positive and negative correlations.

Next, we attempted to determine the proportion of immune cells in the microenvironment of the lesion. To do this, we used the ESTIMATE (Hu et al., 2019) algorithm to estimate the stromal score, immune score and ESTIMATE score of each sample based on expression data from the two diseases. The different *p*-values among subgroups were calculated by between-group Wilcox tests; then, we plotted a violin plot.

### 2.7 Final key genes’s PPI network construction

PPI analysis of the final key genes and their interacting genes was performed in the GeneMANIA (Warde-Farley et al., 2010) database to predict colocalization, shared protein domains, co-expression, prediction, and correlations between pathways.

### 2.8 TF-miRNA-target network analysis

In order to further understand the regulatory mechanisms associated with the key genes, we next constructed a TF-miRNA-target network for the key genes. The interrelated miRNAs of DFU and CLE were retrieved from the HMDD V3.0 database (Huang et al., 2019) (http://www.cuilab.cn/hmdd); then, we focused on the intersection of the data to identify the miRNAs that were common for the two diseases. The miRWalk database [R] (http://129.206.7.150/) was used to predict the miRNAs for the key genes, select miRNAs that also existed in miRDB, and then identify the intersection. Next, we used Cytoscape (Shannon et al., 2003) (version 3.4.0, http://chianti.ucsd.edu/cytoscape-3.4.0/) software to construct a network diagram. Subsequently, we used DIANA-miRPath v3.0 (Vlachos et al., 2015) (http://www.microrna.gr/miRPathv3/) software to perform shared miRNA KEGG pathway analysis. Next, we used the online database TRRUST V2.0 (Han et al., 2018) (Transcriptional Regulatory Relationships Unraveled by Sentence-based Text mining, http://www.grnpedia.org/trrust/), set the species to “human”, and predicted the upstream transcription factors of our set of key genes. Then, we combined the targeting relationship between miRNAs and key genes and used Cytoscape software to construct a TF-miRNA-target network.

### 2.9 Predictive analysis of key gene drugs

Finally, we used the DGIdb gene-drug interaction database (Cotto et al., 2018) to search for therapeutic drugs that could target the key genes and investigate whether there are drugs that could target the key genes to treat DFU and CLE. Cytoscape software was used the visualize the drug-gene interaction network.

## 3 Results

### 3.1 Identification and function of DEGs

**FIGURE 1:**
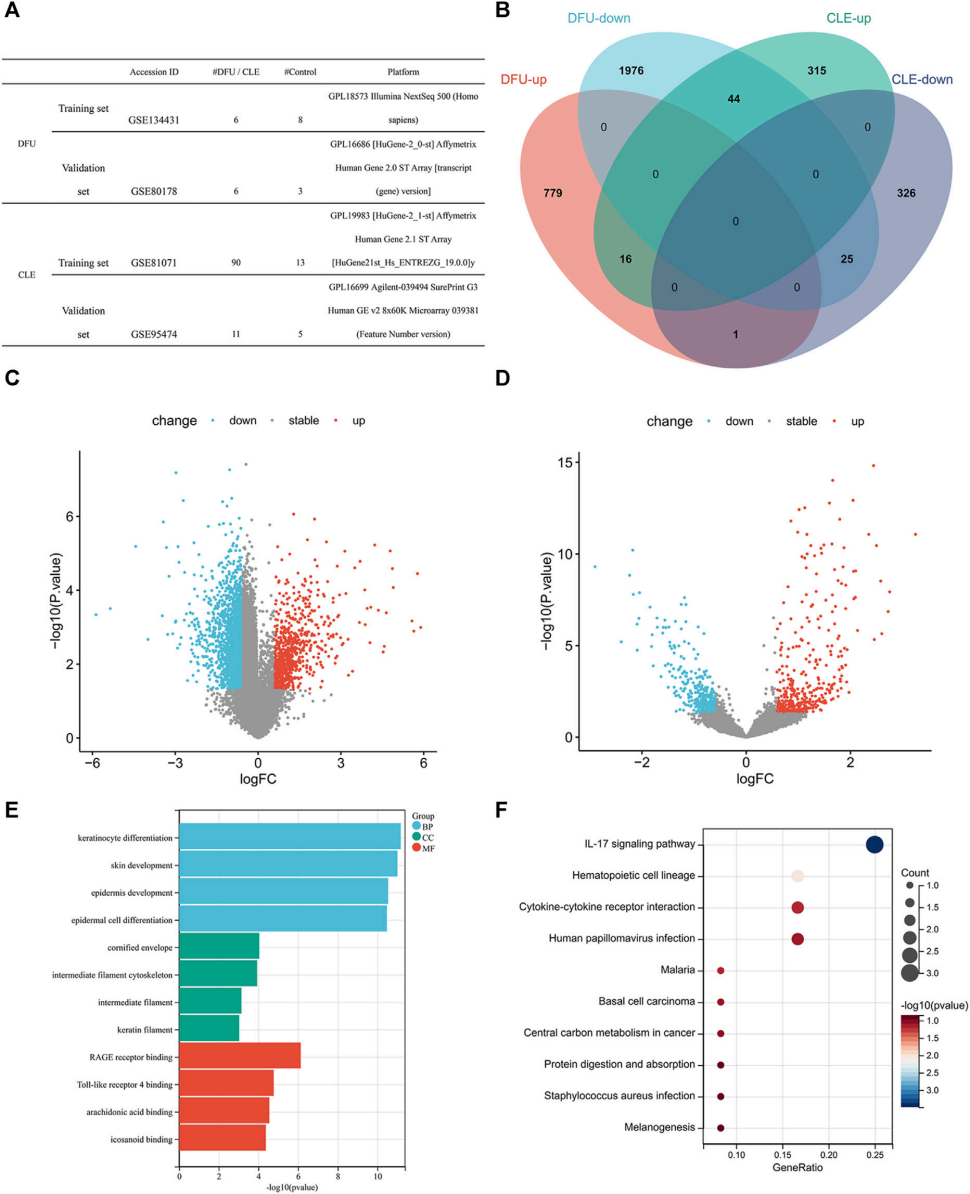
**(A)** Data-set information. **(B)** Venn diagram showing the intersection of differentially expressed genes for the two diseases in training set. **(C)** Volcano plot of differentially expressed genes in diabetic wounds in training set (red indicates upregulated genes, blue indicates downregulated genes, and gray indicates non-significant gene differences). **(D)** Volcano plot of differentially expressed genes in cutaneous lupus erythematosus in training set (red indicates upregulated genes, blue indicates downregulated genes, and gray indicates non-significant gene differences). **(E)** GO enrichment bar chart showing common differentially expressed genes. **(F)** KEGG enrichment bubble chart showing common differentially expressed genes.

Information provided by the dataset used in this analysis is shown in Figure 1A. According to our specific threshold, we identified 796 upregulated and 2045 downregulated genes for DFU, and a total of 375 upregulated genes and 352 downregulated genes for CLE; detailed information is provided in Supplementary Table S2. The identified DEGs are shown as volcano plots in Figures 1C, D. A total of 16 consistently upregulated genes and 25 consistently downregulated genes were identified by considering the intersection of consistent genes between the two diseases, as shown in Figure 1B. See Supplementary Table S2 for further details.

In order to further understand the biological significance of these DEGs, we performed GO functional analysis and KEGG pathway enrichment analysis on the common DEGs. In terms of biological processes, GO analysis showed that the DEGs were mostly related to keratinocyte differentiation, epidermis and skin development, and epidermal cell differentiation. In terms of cellular components, GO analysis showed that the DEGs were mostly related to the cornified envelope, intermediate filament cytoskeleton, intermediate filament and keratin filament. GO analysis also identified several molecular functions for the DEGs, including RAGE receptor binding, Toll-like receptor 4 binding, arachidonic acid binding, and icosanoid binding (Figure 1E; Supplementary Table S3), all of which play important roles in the occurrence and development of excessive/chronic inflammation. Furthermore, KEGG analysis showed that the DEGs were mainly enriched in the IL-17 signaling pathway and hematopoietic cell lineage (Figure 1F; Supplementary Table S3).

### 3.2 PPI network construction and module analysis

**FIGURE 2:**
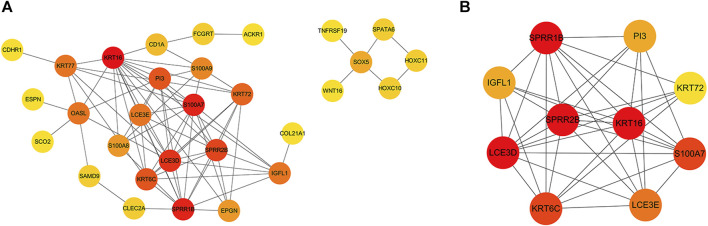
**(A)** PPI network constructed by common differential genes. The gray line represents the interaction between the corresponding proteins of the genes; the redder the color, the more critical the gene is. **(B)** The key sub-module in the PPI analysis with a score of 8.444. The redder color indicates an important interaction in the development of the disease.

Next, we constructed a PPI network for the common DEGs, as shown in Figure 2A. We identified 78 interaction pairs featuring 30 genes and proteins, thus indicating close interaction between these genes; these interactions may play an important role in disease progression (Supplementary Table S4). Furthermore, we analyzed sub-modules of the PPI network and identified the most specific sub-module (Nodes with a high topological score are considered as important nodes in the network, proteins in the submodules because of the core proteins in the PPI network), featuring a total of 10 genes (with a score of 8.444): *SPRR1B* (small proline rich protein 1B), *SPRR2E* (small proline rich protein 2E), *KRT16* (keratin 16), *S100A7* (S100 calcium binding protein A7)*, KRT6C* (keratin 6C)*, LCE3D* (late cornified envelope 3D)*, PI3* (peptidase inhibitor 3)*, KRT72* (keratin 72)*, LCE3E* (late cornified envelope 3E) *and IGFL1* (IGF like family member 1). The red color of the first six proteins is deeper, suggesting that these proteins play an important interaction in the development of the disease. Network diagrams for each sub-module are shown in Figure 2B.

### 3.3 Identification of key genes

**FIGURE 3:**
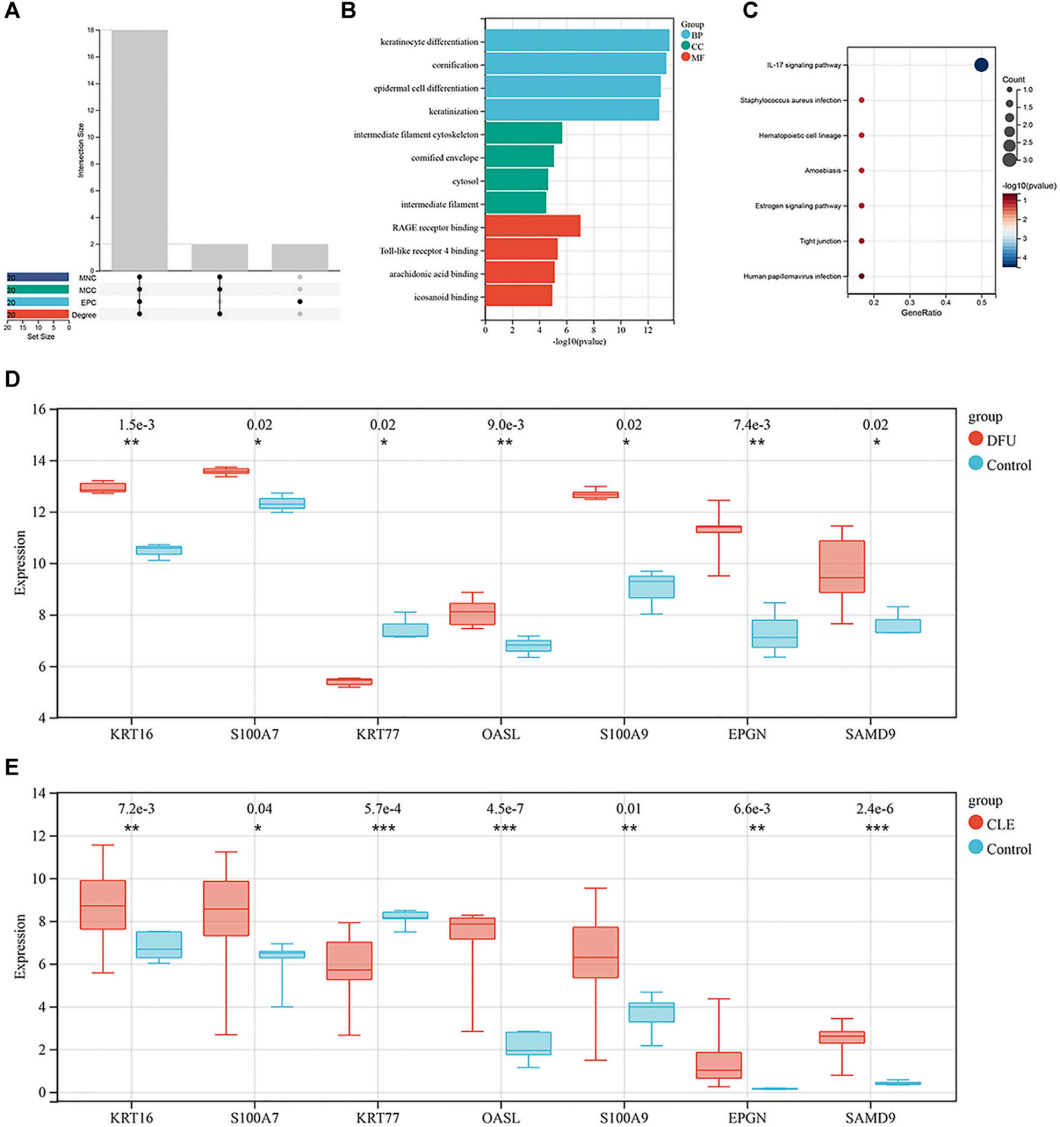
**(A)** Intersections of each topological property ranking the top 20 nodes in the PPI network. Where the abscissa are Edge Percolated Component (EPC), Maximal Clique Centrality (MCC) and Maximum Neighborhood Component (MNC) **(B)** Bar chart showing the key genes identified by GO enrichment. **(C)** Bubble chart showing the key genes identified by KEGG enrichment. **(D)** Box plot showing the distribution of expression levels for seven key genes in the set of DFU validation samples. **(E)** Box plot showing the distribution of expression levels for the seven key genes in the set of CLE validation samples.

The topological properties of the nodes were analyzed using the cytoNCA plug-in. We selected the top 20 genes under each attribute for intersection, as shown in Figure 3A. Finally, 18 genes were identified in the top 20 genes of each attribute; these were considered key genes. Next, we performed GO functional analysis (Figure 3B) and KEGG pathway analysis (Figure 3C) for the shared key genes. GO results showed that the key genes were mainly related to the keratinization and differentiation of skin-related cells, intermediate filament cytoskeleton, and RAGE receptor binding. IL-17 signaling pathway was also significantly enriched, as determined by KEGG pathway enrichment. The enriched genes were *S100A7, S100A9* and *S100A8*; see Supplementary Table S5 for details.

In order to verify the expression levels of the key genes, we generated box plots showing the distribution of expression levels for the 18 key genes by mining a diabetic wound training set and cutaneous lupus erythematosus in the training set. As shown in Figures 3D, E, respectively, a total of seven genes were successfully verified; of these, *KRT16, S100A7, OASL, S100A9, EPGN* and *SAMD9* were upregulated, and *KRT77* was downregulated. Of these, *KRT16* and *S100A7* were also present in the sub-modules analyzed by MCODE.

### 3.4 Association between key gene and immune infiltration

**FIGURE 4:**
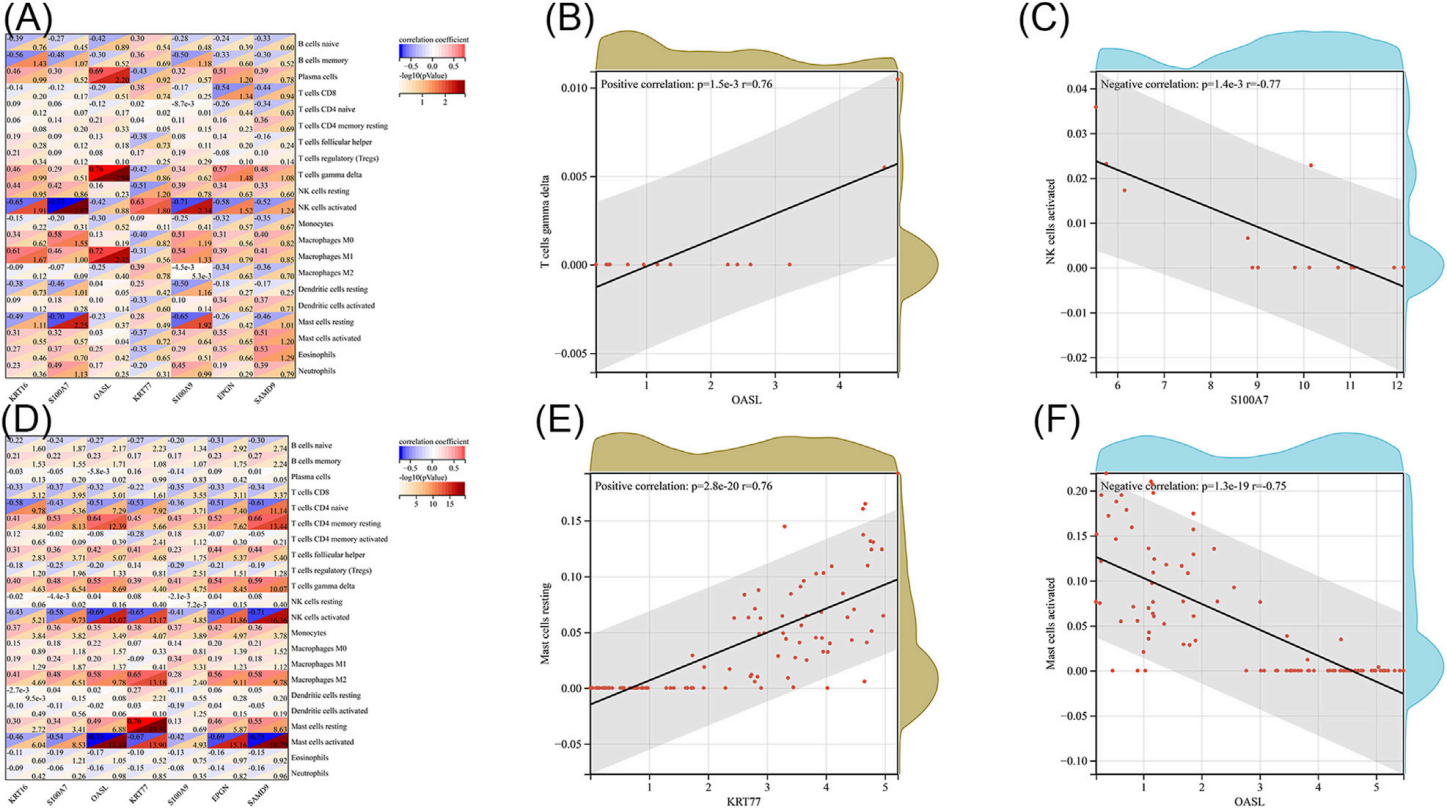
**(A)** Heat map showing the correlation between key genes and immune cells in diabetic wounds. **(B)** Scatter plot of OASL and gamma delta T cells in diabetic wounds. **(C)** Scatter plot of S100A7 and activated NK cells in diabetic wounds. **(D)** Heat map showing the correlation between key genes and immune cells in systemic lupus erythematosus. **(E)** Scatter plot of KRT77 and resting mast cells resting in CLE. **(F)** Scatter plot of OASL and activated mast cells in CLE.

Next, we investigated the association between immune cell infiltration and our list of key genes. We used the CIBERSORT algorithm and the LM22 gene set to determine training data sets for DFU and CLE; this gave us the proportions of 22 different types of immune cells in each sample (Supplementary Table S6). We calculated the correlation coefficients and *p*-values between each key memory and each immune cell by Pearson’s correlation. The correlation heat map for DFU is shown in Figure 4A. As shown in Figure 4B, the highest positive correlation was detected between *OASL* and gamma delta T cells, with a correlation of 0.76. The highest negative correlation was identified between *S100A7* and activated NK cells, with a correlation of −0.77, as shown in Figure 4C. Figure 4D shows the correlation heat map for CLE. The highest positive correlation was identified between *KRT77* and resting mast cells, with a correlation coefficient of 0.76 (Figure 4E). The highest negative correlation was identified between *OASL* and activated mast cells, and between *SAMD9* and activated mast cells, both with a correlation coefficient of −0.75, as shown in Figure 4F.

### 3.5 PPI network construction of key genes

**FIGURE 5:**
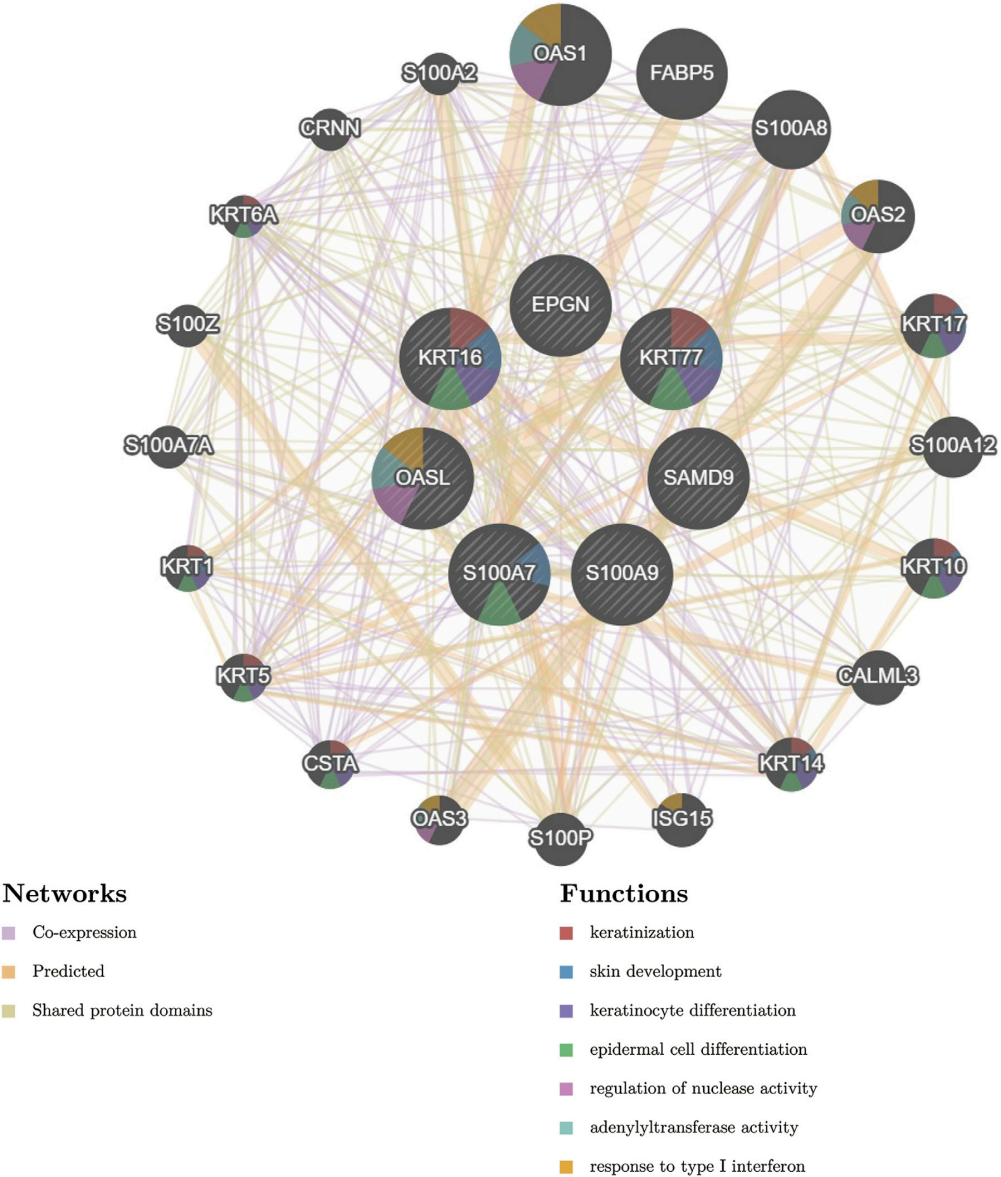
PPI network of key genes and their interacting genes. (Firstly, the related miRNA were searched in HMDD V3.0 database, and the key gene miRNA were predicted by miRWalk database, and the intersection of the two was taken. Then, we used DIANA-miRPath for pathway analysis, combined with the targeting relationship between MIRNA and key genes. Finally, we used Cytoscape software to construct a network diagram).

Next, we constructed PPI networks for the DEGs with the aim of identifying the close relationship between these genes and identifying significant key genes by topological analysis of the nodes of the PPI network of DEGs. In order to predict the co-localization, shared protein domains and co-expression of these key genes, and to predict the correlation between key gene pathways, we used the GeneMANIA database to conduct PPI analysis of the final key genes and interaction genes, as shown in Figure 5. The pathways associated with these seven key genes were closely related to skin development, especially keratinocyte differentiation, as well as to the regulation of nuclease activity, adenylyl transferase activity, and response to type I interferon.

### 3.6 TF-miRNA regulatory network analysis

**FIGURE 6:**
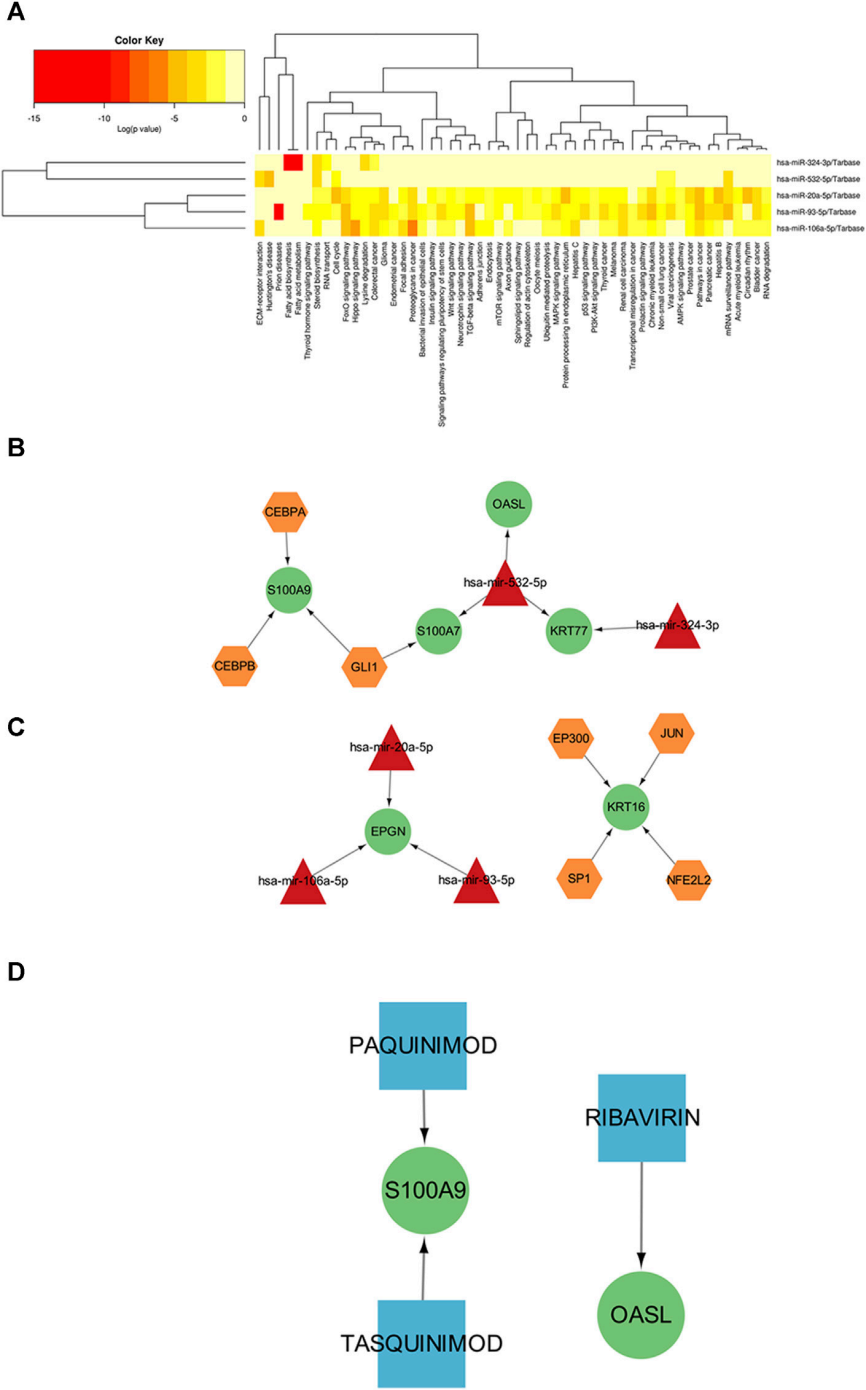
**(A)** KEGG results showing the significant enrichment of common miRNAs for the two diseases (the redder the color, the greater the significance). **(B)** Interaction diagram showing the miRNA-target gene network relationship. **(C)** TF-miRNA-target network (yellow hexagon represents transcription factor (TFs), red triangle represents miRNAs, green circle represents key genes). **(D)** Drug-target network (blue squares represent drugs, green circles represent key genes).

Transcription factors (TF) and miRNAs, as key factors in transcriptional and post-transcriptional regulation, play an important role in gene expression regulation of cells. In order to further elucidate the functions of common mirnas, we constructed Gene-TF-MIRNA networks to more systematically understand the regulatory pathways of key genes and provide important clues for the occurrence and development of diseases and targeted therapy. Based on the HMDD database, we retrieved miRNAs that were related to cutaneous lupus erythematosus, but failed to identify any miRNAs that were specifically related to diabetic wounds. Then, we intersected the miRNAs related to cutaneous lupus erythematosus and the miRNAs predicted by the common key genes. Next, we performed functional pathway analysis for the common miRNAs; the results are shown in Figure 6A. The most significant function of the common miRNAs was the biosynthesis of fatty acids. Diabetes is a disorder of glucose metabolism in the blood; fatty acid biosynthesis is also an aspect of glucose metabolism in the blood. This provides evidence that the common miRNAs are also involved in the development of DFU. Then, we constructed a network diagram based on miRNAs and target genes, as shown in Figure 6B, including hsa-mir-532-5p (targeting *S100A7, OASL, KRT77*), hsa-mir-324-3p (targeting *KRT77*) and hsa-mir-20a-5p/hsa-mir-106a-5p/hsa-mir-93a-5p (targeting *EPGN*). Finally, we performed upstream transcription factor prediction analysis for the seven key genes. We constructed a TF-miRNA-target network based on the combined miRNA information. A total of seven transcription factors (CEBPA, CEBPB, GLI1, EP300, JUN, SP1, NFE2L2) were predicted, as shown in Figure 6C.

### 3.7 Drug prediction of key gene

Finally, we used the DGIdb gene-drug interaction database to identify therapeutic drugs that could target the key genes. The predicted relationship between drugs and genes is detailed in Supplementary Material 8 and the network relationship is shown in Figure 6D. The results showed that Paquinimod, Tasquinimod and Ribavirin are potential drugs that could target *S100A9* and *OASL*. Paquitimod and Tasquinimod both are immunomodulatory compounds targeting S100A9, which inhibits the pro-inflammatory cytokine response of monocytes by blocking the binding of S100A9 to TLR4 and RAGE (Cesaro et al., 2012; Bresnick, 2018; Le Bagge et al., 2020). Ribavirin is a guanosine analogitic, which is currently used in the treatment of hepatitis virus infection due to its extensive antiviral activity. It is mainly used in combination with polyethylene glycol to inhibit the expression of OASL, IFITI, CXCL10 and other cytokines (Brodsky et al., 2007; Su et al., 2008; Boros and Vécsei, 2020).

## 4 Discussion

Skin lesions are common symptoms of both DFU and CLE, and present as persistent and non-healing wounds. However, few studies have investigated the relationship and differences between the pathogenesis and progression of DFU and CLE, especially in terms of specific cellular and molecular mechanisms. In this study, we used a bioinformatic approach to identify common DEGs between these two diseases. We also identified potential key genes involved in the interaction between DFU and CLE, including 16 upregulated genes and 25 downregulated genes. After validation of the second dataset, 7 key genes with consistent differential expression trends were finally identified in the two diseases. Of these, KRT16, S100A7, OASL, S100A9, EPGN and SAMD9 were upregulated while KRT77 was downregulated.

*S100A7* first came to public attention because it was identified as a secreted protein that was over-expressed in psoriatic skin (Madsen et al., 1991). Both DFU and psoriasis are related to abnormal keratinocyte functionality (Granata et al., 2019). DFU patients exhibit slow re-epithelialization of keratinocytes along with a chronic inflammatory environment and healing disorders at lesion sites (Yang et al., 2022; Fu et al., 2023). Previous studies showed that the *S100A7* mouse model of psoriasis exhibited lesions that were characterized by leukocyte inflammation (Webb et al., 2005). Interestingly, the failure of DFU wound healing is associated with a reduction in the number of M2 reparative macrophages at the wound site (Aitcheson et al., 2021). In addition, *S100A7* was shown to be significantly upregulated in the skin ulcers of patients with DFU (Shaorong et al., 2023). As mentioned earlier, the apoptosis of keratinocytes is also closely related to the pathogenesis of CLE skin lesions. Keratinocytes may also participate in lupus skin lesions by releasing proinflammatory cytokines (Doersch et al., 2017). We identified *S100A7* as a key gene for both DFU and CLE; thus, this particular gene is associated with two different types of skin damage. Previous studies have shown that the abnormal morphology and functionality of keratinocytes caused by the upregulation of *S100A7* may be involved in the skin damage experienced by patients with DFU and CLE. In a hyperglycemic environment, *S100A9* not only activates the proinflammatory activity of macrophages via the RAGE pathway; it also induces the secretion of proinflammatory cytokines in macrophages via the NF-kB pathway (Kawakami et al., 2020). *S100A9* is also a relevant marker for CLE patients (Soyfoo et al., 2009) and is expressed in important immune cells such as monocytes, neutrophils and B cells during the CLE inflammatory response (Lood et al., 2011). Therefore, as a known proinflammatory factor, *S100A9* also seems to play a role in perpetuating and extending inflammation in DFU and CLE.

Other key genes also suggest avenues to explore. The keratin gene *KRT77* plays a key role in the transcriptional programming of early epidermal maturation; the expression levels of this gene are known to be suppressed during normal epidermal differentiation and subsequent development (Sevilla et al., 2010), our analysis confirmed these previous findings. *EPGN* is the ligand for epidermal growth factor receptor (EGFR) (Singh et al., 2016); the upregulation of *EPGN* affects EGFR homeostasis and leads to hyperplasia of the sebaceous gland in mice (Dahlhoff et al., 2010). Unfortunately, there is an insufficient body of data to confirm the effect of *EPGN* upregulation on the occurrence and development of CLE and DFU. Further research is required to address this issue. *SADM9* is a gene located on human chromatid 7 that exhibits anti-tumor and anti-virus activities (Inaba et al., 2018). Some researchers have found that there are domains related to inflammation and apoptosis in the *SADM9* protein (Mekhedov et al., 2017). The main role of OAS family is regarded as an immunomodulator, and *OASL* level is associated with autoimmune diseases and chronic infections (Choi et al., 2015). OASL expression is upregulated in SLE patients (Gao et al., 2020), and OASL expression is present in type I IFN response in CLE patients and type I diabetic patients (Sarkar et al., 2018; Pedersen et al., 2021). Moreover, *OASL* regulates IFN activation to promote abnormal proliferation and differentiation of keratinocytes in psoriatic lesions (Huang et al., 2022). Therefore, we tentatively hypothesize that the upregulation of *SADM9* and OASL expression in response to IFN signal might be involved in the development and persistence of inflammation and in both DFU and CLE.

Our analysis also demonstrated that the key genes identified were mainly related to the keratinization and differentiation of skin cells and inflammatory pathway-related receptors such as rage receptor binding and TLR4 receptor binding. On the one hand, the physiological environment of hyperglycemia in diabetic patients stimulates the AGE-RAGE pathway, thus triggering a persistent inflammatory response that inhibits the healing of ulcers in DFU patients (Song et al., 2022). The inflammatory damage caused by oxidative stress and the AGEs-RAGE pathway has also been detected in CLE patients (Martens et al., 2012). On the other hand, TLR4 can induce the production of various proinflammatory cytokines and is also involved in inflammatory responses in pancreatic islets, fat, liver and kidney tissues, all of which have been implicated in the development of diabetes and systemic lupus erythematosus (Wada and Makino, 2016; Zhang et al., 2016). Furthermore, activation of the TLR4 pathway by a hyperglycemic environment is known to impair wound healing in mice (Portou et al., 2020). Collectively, our results suggest that both RAGE and TLR4 are involved in the inflammatory response in CLE and DFU patients. Arachidonic acid and eicosanoid acid are fatty acids related to inflammation. The metabolism of arachidonic acid is abnormally accelerated in diabetic patients and damages pancreatic β cells exposed to the inflammatory environment caused by arachidonic acid and eicosanoid acid metabolism (Halushka et al., 1985; Das, 2013). Similarly, the circulating composition of inflammation-associated fatty acids has also been shown to be altered in patients with CLE; this was coincident with a significant increase in the plasma levels of arachidonic acid (Aghdassi et al., 2011). The enrichment results of our experiments identified key genes that were related to arachidonic acid and eicosanoid receptor binding. Collectively, these data provide evidence that DFU and CLE have similar pathological processes.

According to previous studies, DFU and CLE have similar immune manifestations in skin lesions. Patients with DFU are also known to have higher levels of many inflammatory cytokines, including IL-8, TNFα, and CRP (Tecilazich et al., 2013). He levels of cytokines (IL-1β, TNF-α, IFN-γ and IL-10) are also increased in CLE patients (McCarthy et al., 2014). Interestingly, despite similar expression differences of these key genes in both diseases, their correlation with immune cell responses exhibits divergent, and even opposing trends. In Diabetic Foot Ulcers (DFU), OASL showed a positive correlation with gamma delta T cells in DFU, whereas negatively correlated with activated mast cells in CLE. This observed variance in correlation across different diseases may reflect the distinct pathological mechanisms underlying DFU and CLE, as well as the differential responses of the immune system in varying disease contexts. DFU, being a chronic wound associated with metabolic disease, likely presents a fundamentally different immunological environment compared to CLE, an autoimmune skin condition, particularly in terms of immune cell activity and regulation.

Finally, we found that paquinimod and taquinmod can target *S100A9* while ribavirin can target *OASL*, as demonstrated by a DGIdb gene-drug interaction database. Paquinimod has shown a similar efficacy to the currently used immunosuppressants prednisolone and mycophenolate mofetil in mice with lupus (Bengtsson et al., 2012). In the skin models of systemic sclerosis and psoriasis mice, which are also autoimmune diseases, the use of paquinimod targeting S100A9 reduced skin fibrosis and improved skin inflammation (Stenström et al., 2016; Khaleel and Zalzala, 2023; Silva De Melo et al., 2023). Such an autoimmune suppressant also prevented the development of type 1 diabetes in mice (Tahvili et al., 2018; Le Bagge et al., 2020). All of this evidence suggests the availability of pacquimod as a potential therapeutic agent for DFU and CLE. The pharmacological activity of taquinmod is more associated with anti-vascular and anti-prostate cancer effects (Isaacs et al., 2006; Olsson et al., 2010; Boros and Vécsei, 2020). In the pathological environment of high glucose, taquinmod inhibits proliferation, migration and lumen formation of human retinal endothelial cells (Jin et al., 2022). Ribavirin, as an antiviral drug, is considered as a potential candidate for the treatment of HFMD(Leung et al., 2022). Paradoxically, ribavirin combined with cyclophosphamide or interferon has a high risk of skin and appendage adverse reactions (rash, cutaneous sarcoidosis, etc.) (Kato et al., 2021; Zheng et al., 2022).The efficacy of ribavirin in DFU and CLE remains to be further clarified.

However, there are some limitations of our study that need to be considered. First, the specific role of the two keratin genes in the disease process has yet to be elucidated. The expression of *KRT77* was upregulated during epidermal development, while *KRT77* was downregulated in the DFU and CLE datasets we analyzed. More data are needed to confirm that *KRT77* downregulation and *KRT16* upregulation are associated with the pathological changes of these two diseases. Further *in vitro* studies are now needed to better explain the mechanisms by which *S100A9, EPGM, SADM9* and *OASL* can cause skin lesions in DFU and CLE and the therapeutic effects of paquinimod and taquinmod on DFU and CLE. Finally, there is not enough evidence to prove that ribavirin can treat the pathological state caused by the abnormal expression of *OASL*; this still needs to be verified in future clinical trials.

## 5 Conclusion

In summary, we identified a set of DEGs shared by DFU and CLE from datasets in public databases. Enrichment analysis revealed that the genes common to DFU and CLE were related to pathological changes and inflammation of the epidermis. PPI network construction identified seven common key genes, including *KRT16, S100A7, OASL, S100A9, EPGN SAMD9* and *KRT77.* A quite different patterns of immune cell infiltration indicated that similar final inflammatory mechanism could be associated with different upstream immunopathological mechanisms. In addition, TF-miRNA regulatory network analysis and drug prediction provided a positive indicative role in identifying targets for subsequent research and treatments.

## Data Availability

The datasets presented in this study can be found in online repositories. The names of the repository/repositories and accession number(s) can be found in the article/Supplementary Material.
